# Metabolic cues are the main drivers for the differentiation of chloroplasts into chromoplasts and other plastid types

**DOI:** 10.1093/jxb/erag235

**Published:** 2026-05-20

**Authors:** Manuel Rodriguez-Concepcion, Shan Lu

**Affiliations:** Institute for Plant Molecular and Cell Biology (IBMCP), CSIC-Universitat Politècnica de València, València 46022, Spain; State Key Laboratory of Pharmaceutical Biotechnology, School of Life Sciences, Nanjing University, Nanjing 210023, China; Max Planck Institute for Molecular Plant Physiology, Germany

**Keywords:** Chloroplasts, chromoplasts, differentiation, plastids, storage, transition, ultrastructure

## Abstract

Plastids show an astounding degree of functional and structural plasticity, ranging from photosynthetic chloroplasts with thylakoids and grana to non-photosynthetic storage organelles such as chromoplasts, which are specialized in the synthesis and sequestration of carotenoid pigments in plastoglobules, crystalline structures, and membrane sacs. Transitions between different plastid types involve extensive rewiring of nuclear gene expression to support the concomitant changes in the proteome and the ultrastructure of plastids. However, the signals triggering the underlying molecular mechanisms are not well understood. In this review, we focus on the chloroplast-to-chromoplast differentiation process in both natural and synthetic (genetically engineered) systems. Using tomato as a model, the review outlines the sequential molecular, structural, and metabolic events driving chromoplastogenesis, including chlorophyll degradation, carotenoid biosynthesis, proteome remodeling, and plastid ultrastructure reorganization. Synthetic systems mimicking this process reveal that sufficient loss of chloroplast identity followed by carotenoid overaccumulation can independently trigger chromoplast formation in plant species and tissues that do not naturally differentiate chromoplasts. These insights highlight metabolic cues as primary drivers of plastid differentiation and adaptation.

## Teaser

We revise the chloroplast-to-chromoplast differentiation process in natural and engineered systems as a model to understand plastid interconversions. Emerging evidence indicates that metabolic cues are the main drivers for changing plastid identity.

## Introduction

Plastids are one of the most defining features of the plant kingdom. They are organelles originally acquired by eukaryotic cells through endosymbiosis with a cyanobacterial ancestor ∼1.6 billion years ago (Ponce-Toledo *et al.*, 2017; Bhide, 2025). This primary engulfment and integration of a cyanobacterium into the eukaryotic cell occurred in the ancestor of Archaeplastida, a eukaryotic supergroup that includes higher plants. Chloroplasts are the cyanobacteria-derived photosynthetic organelles that are currently present in plant cells. However, more recent endosymbiotic episodes have occurred independently (Bhide, 2025). Notable examples are the sausage-shaped photosynthetic organelles known as chromatophores present in the free-living freshwater amoeba *Paulinella chromatophora* (Bodył *et al.*, 2012) and the nitrogen-fixing nitroplast found in the microalga *Braarudosphaera bigelowii* (Coale *et al.*, 2024).

As the endosymbiotic event that originated higher plant chloroplasts evolved, plastids coordinated their division with the host cell cycle and transferred a substantial part of their ancestral genome to the nuclear genome of the host (Hashimoto, 2003; Herrmann *et al.*, 2003). Only about a hundred genes are retained in the current plastid genome (plastome), and most of these genes encode proteins of the photosynthesis apparatus and components of the genetic system needed for plastome gene expression (Sato *et al.*, 1999). The vast majority of proteins required for plastid biogenesis and function are encoded by the nuclear genome and need to be imported into plastids when required. Nuclear-encoded plastid proteins usually contain a variable N-terminal sequence that targets them to plastids after their synthesis in the cytosol. Plastid import of such proteins involves a sophisticated machinery located in the envelope membranes. Once inside the plastid, the N-terminal transit sequence is cleaved in the stroma, and intraplastidic transport systems target proteins to their final destination (Sun and Jarvis, 2023). A diverse array of chemical compounds released by the plastid (referred to as retrograde signals) orchestrate chloroplast biogenesis and adjust nuclear gene expression to the physiological state and requirements of plastids (Calderon and Strand, 2021; Sierra *et al.*, 2023; Loudya *et al.*, 2024).

**Fig. 1.**
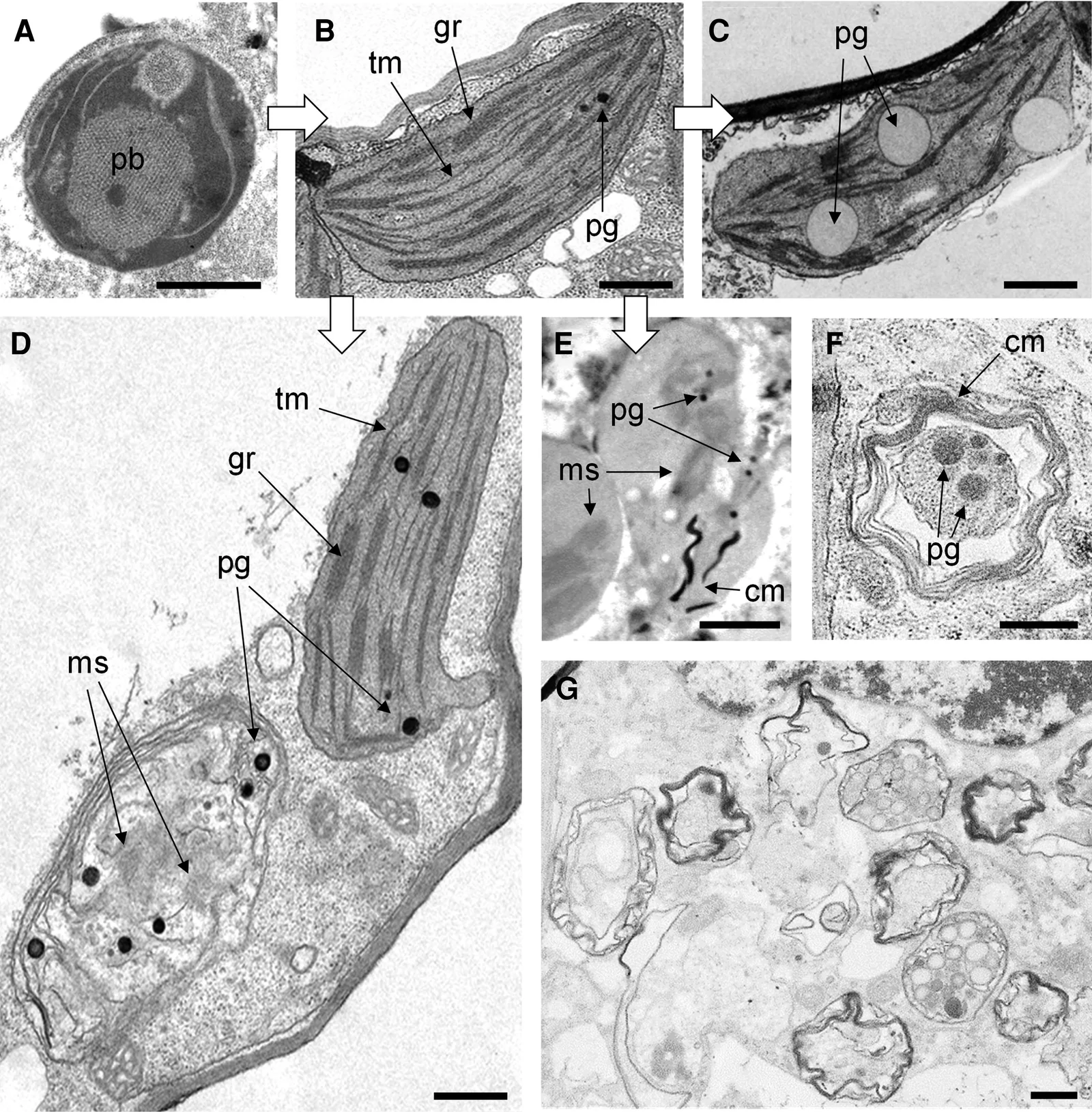
TEM pictures of representative plastid types. (A) *Arabidopsis thaliana* etioplast (from the cotyledon of a dark-grown seedling). (B) *A. thaliana* leaf chloroplast. (C) *A. thaliana* gerontoplast from a dark-incubated senescent leaf. (D) Chloroplast and synthetic chromoplast in the same cell of a crtB-producing *Nicotiana benthamiana* leaf. (E) Chromogerontoplast from a SGR-producing and dark-incubated *N. benthamiana* leaf. (F) Tomato ripe fruit chromoplast. (G) Diversity of chromoplasts in the same pericarp cell of a ripe tomato. Images were taken by Pablo Perez-Colao (A), Briardo Llorente (B, C, D), Hui Zheng (E), and Lucio D’Andrea (F. G). Bar, 1 µm; cm, crystal membrane; gr, granum; ms, membrane sac; pb, prolamellar body; pg, plastoglobule; tm, thylakoid membrane.

During evolution, chloroplasts radiated into a broader family of plastids that lost their photosynthetic function and instead synthesized and accumulated different metabolites (de Vries *et al.*, 2016; Sadali *et al.*, 2019; Choi *et al.*, 2021; Sierra *et al.*, 2023) (Fig. 1). Proplastids, which are small undifferentiated precursor organelles mainly present in meristematic and reproductive tissues (Choi *et al.*, 2021; Sierra *et al.*, 2023), give rise to chloroplasts or other plastid types depending on developmental (e.g. tissue) and environmental (e.g. light) cues. For example, the cotyledons of seedlings germinated in darkness (e.g. underground) differentiate etioplasts (Fig. 1A), but produce chloroplasts if germination takes place in the light (Fig. 1B). When etiolated seedlings emerge from the soil and are illuminated, their cotyledon etioplasts differentiate into chloroplasts, whereas chloroplasts are transformed into gerontoplasts when photosynthetic tissues such as leaves enter senescence (Fig. 1C). Plastid types found in non-photosynthetic tissues of light-grown plants include leucoplasts (a generic term that includes plastids without color) and chromoplasts (plastids specialized in the accumulation of carotenoid pigments). Depending on the tissue and the plant species, leucoplasts may specialize in the accumulation of particular metabolites and become amyloplasts (with starch granules), proteinoplasts (with protein bodies and crystals), or elaioplasts (with fatty acids and other lipids in plastoglobules). Other specialized plastids are phenyloplasts (phenol-enriched), xyloplasts (dedicated to the synthesis of monolignol precursors), and tannoplasts (which accumulate tannins), among others (Choi *et al.*, 2021; Sierra *et al.*, 2023).

Notably, plastid differentiation is not a terminal process, as plastids can dynamically interconvert and even co-exist with other plastid types in the same cell (Fig. 1D). Furthermore, intermediate plastid types such as chloroamyloplasts, chlorochromoplasts, amylochromoplasts, or chromogerontoplasts (Fig. 1E) have been reported, illustrating an astounding degree of plasticity (Choi *et al.*, 2021; Sierra *et al.*, 2023; Zheng *et al.*, 2025). Despite the fundamental importance of plastid differentiation for plant growth and human health, we know very little about the molecular mechanisms behind plastid transitions. In this review, we focus on the chloroplast-to-chromoplast differentiation process, which is one of the best studied plastid transitions as it is associated with economically relevant issues such as pigmentation, fruit ripening, and nutritional enrichment.

## Chromoplasts and carotenoids

Chromoplasts are generally defined as plastids specialized in the production and accumulation of carotenoid pigments (Box 1). Carotenoids can be found in most plastid types but they are most abundant in chloroplasts and chromoplasts. In chloroplasts, they play essential roles in photosynthesis and photoprotection (Hashimoto *et al.*, 2016; Rodriguez-Concepcion *et al.*, 2018; Sun *et al.*, 2018). In chromoplasts, carotenoids provide yellow to red pigmentation to non-photosynthetic tissues. For example, they contribute to pollination by coloring flowers and to seed dispersal by informing animals when fruits are ripe. Besides this ecological role, carotenoid pigments in vegetables enhance consumer acceptance and improve nutritional value as precursors for the production of retinoids (including vitamin A) and health-promoting metabolites (Rodriguez-Concepcion *et al.*, 2018). Furthermore, the use of carotenoids as natural pigments is widespread in the agrofood and cosmetic industries. As chromoplasts develop sequestering structures that contribute to accumulate high levels of carotenoids and to protect them from degradation, recent attention has been centered on investigating how chromoplasts are made, with the eventual goal of improving sink capacity in plant tissues for carotenoid biofortification of crops (Torres-Montilla and Rodriguez-Concepcion, 2021).

Box 1. **What is a chromoplast?**

Chromoplasts are generally defined as plastids specialized in the production and accumulation of carotenoid pigments. However, they are not a homogeneous class of plastids but a pleomorphic group of storage organelles with a wide variety of ultrastructural features depending on their carotenoid composition. Based on recent findings, a more precise definition can be made, describing chromoplasts as plastids that:

- are non-photosynthetic;
- show different carotenoid profiles, depending on the tissue or the species;
- sequester carotenoids in specialized membrane, lipoprotein, or crystalline structures;
- differentiate from chloroplasts or other plastid types in response to a sudden enrichment of carotenoids independently of
  ○ the triggering signal (i.e. either genetically determined or caused by external cues), or
  ○ the origin of the carotenoids (i.e. either rescued from pre-existing locations or produced *de novo*).

Despite chloroplasts containing relatively high levels of carotenoids, photosynthetic tissues such as leaves are normally green due to the presence of chlorophylls. In fact, the balance between carotenoids and chlorophylls is tightly controlled in chloroplasts to ensure correct photosynthesis and photoprotection (Li *et al.*, 2025). Carotenoid pigments become visible in leaves when developmental or environmental cues trigger senescence and chlorophylls degrade (Egea *et al.*, 2010). During leaf senescence, chloroplasts are transformed into gerontoplasts as thylakoids disaggregate and derived lipid-rich lipoprotein bodies (plastoglobules) enlarge (Fig. 1C). Unlike the senescence-associated gerontoplasts responsible for the typical yellow, orange, and red colors of autumn leaves, a handful of plant species can reversibly transform their leaf chloroplasts into chromoplasts (Koiwa *et al.*, 1986). However, chloroplast-to-chromoplast differentiation most often occurs during ripening of many fleshy fruits (e.g. tomatoes and peppers) and it is typically irreversible, even though fruit chromoplasts can return to the chloroplast state in some cases (Mesa *et al.*, 2025). Chromoplasts can also derive from other plastid types, such as leucoplasts, and occur in other organs, including petals (e.g. marigold), seeds (e.g. maize), or roots (e.g. carrots) (Egea *et al.*, 2010; Li and Yuan, 2013; Sun *et al.*, 2018; Sadali *et al.*, 2019; Choi *et al.*, 2021; Sierra *et al.*, 2023).

Carotenoids are highly hydrophobic metabolites that can accumulate in free form, in crystals, aggregated, or associated with other molecules (proteins, fatty acids, or sugars) (Rodriguez-Concepcion *et al.*, 2018). The carotenoids typically found in chloroplasts (lutein, β-carotene, and β,β-xanthophylls) are most abundant in thylakoid membranes, associated with proteins in photosynthetic complexes. They can also be found in lower amounts in the hydrophobic environment of envelope membranes or even in small plastoglobules (Rodriguez-Concepcion *et al.*, 2018; Morelli *et al.*, 2023). Unlike chloroplasts, chromoplasts exhibit a high diversity of carotenoid profiles and consequently of storage structures. Although all chromoplast types develop structures to synthesize and deposit large amounts of carotenoids, there is no single key structure unique to all chromoplasts (Li and Yuan, 2013). In fact, chromoplasts are more a heterogeneous group of carotenoid-rich pleomorphic plastids rather than a specific plastid type (Box 1). Based on the morphology of the sites where these pigments are stored, chromoplasts have been classified into globular (i.e. with large plastoglobules), crystalline, membranous, fibrillar, or tubular. However, this classification is an oversimplification as chromoplasts with different storage structures are common (Sadali *et al.*, 2019). For example, chromoplasts in the ripe fruit of tomato (*Solanum lycopersicum*) plants contain both globular and crystalline structures (Fig. 1F). It is unclear whether these structures develop successively or simultaneously, but it is common to find chromoplasts with a diverse range of ultrastructures in the same cell (Fig. 1G). While some structures are associated with particular types of carotenoids, others can contain a wide diversity of carotenoids. For example, plastoglobules can store phytoene and other non-colored carotenoid pathway intermediates as well as β-carotene or xanthophylls such as lutein, whereas crystals are normally formed when lycopene or β-carotene accumulate at very high levels (Nogueira *et al.*, 2013; Sadali *et al.*, 2019; Morelli *et al.*, 2023; Zhu *et al.*, 2024). The envelope that covers crystalline structures shrinks when carotenoid crystals are lost during the dehydration step of sample preparation for TEM, resulting in very characteristic undulating membranes (Fig. 1E–G). Importantly, the type of storage or sequestration structure (e.g. membrane, lipoprotein, crystals, etc.) strongly determines carotenoid bioaccessibility (i.e. how easily they are released from the food matrix during digestion) and bioavailability (i.e. how well they are absorbed by our bodies), making chromoplast carotenoids more easy to take as nutrients than chloroplast carotenoids (Morelli and Rodriguez-Concepcion, 2023).

## How tomato fruits make chromoplasts

**Fig. 2.**
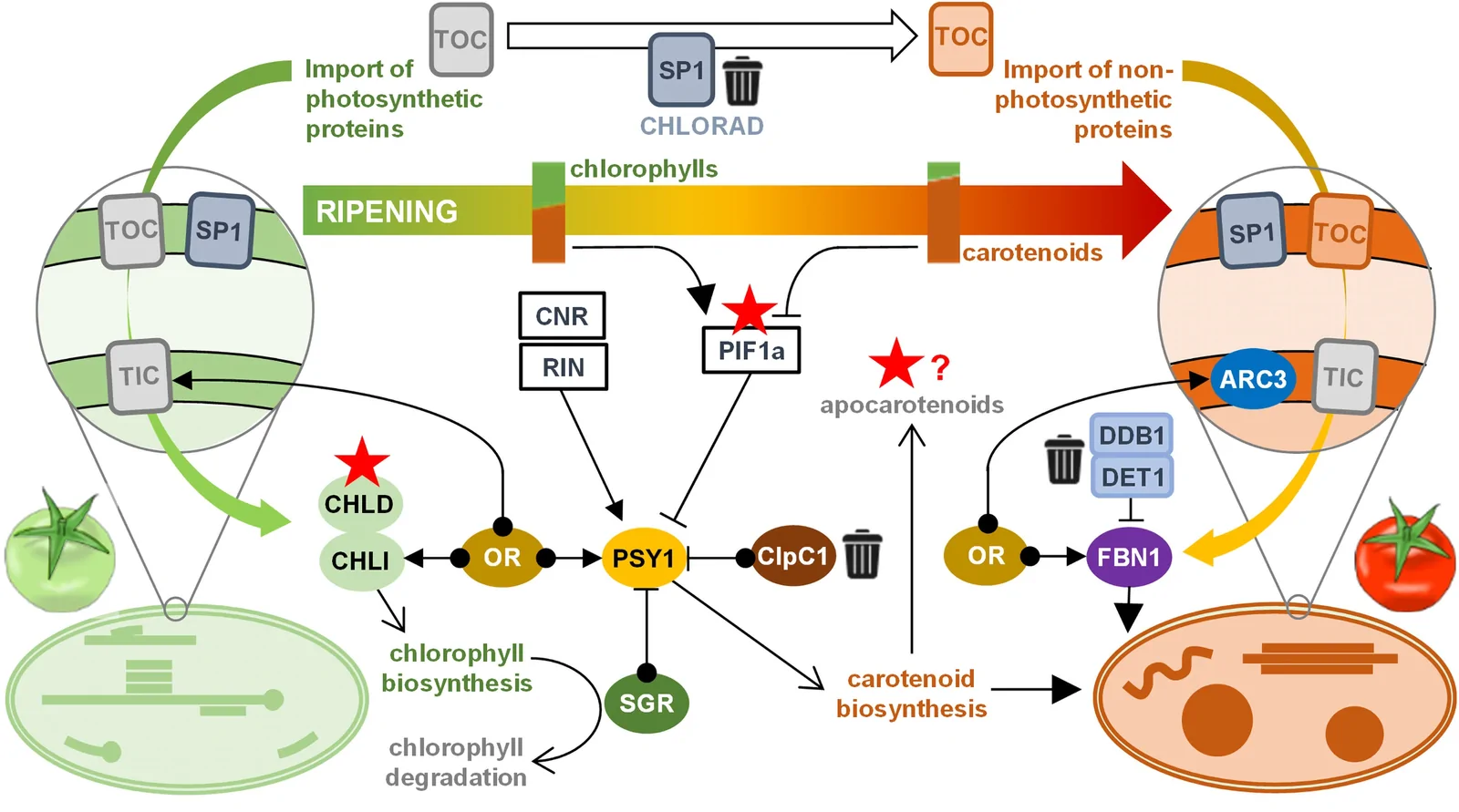
Schematic summary of molecular components involved in the differentiation of chromoplasts from chloroplasts during tomato fruit ripening. Chloroplasts present in mature green fruit (left) differentiate into chromoplasts in ripe fruit (right) during ripening. This conversion involves coordinated changes in gene expression orchestrated by transcription factors (boxed in white) as well as protein import (e.g. the translocon complex of the outer envelope membrane, TOC) and degradation machineries (indicated with a dustbin icon). The progression of ripening is monitored by several sensors (marked with a red star) through light-dependent and retrograde signals. The chaperone OR is a major hub that coordinates different processes by direct interaction with a wide range of proteins (marked with a circle ending with an arrow, for positive regulation, or a bar head for repression). Protein acronyms are spelled out in the text.

The extensive genetic and genomic resources available for tomato, as well as the economic significance of its fruits, have established it as a research model for studying chromoplast development. As the tomato fruit ripens, it undergoes a dramatic and well-characterized transition from green to red, which involves the differentiation of pre-existing chloroplasts into chromoplasts, characterized by the degradation of chlorophylls and a massive accumulation of carotenoids, predominantly lycopene and, to a lower extent, β-carotene. The regulation of tomato fruit chromoplastogenesis occurs at multiple levels. Here we will briefly revise the stages and some of the molecular players involved in the transition (Fig. 2). The presented sequence reflects experimental evidence of temporal progression together with logical causality from the literature, but most stages are highly dynamic and typically overlap.

### The developmental ripening program starts

Fruit ripening is initiated by a developmental program strongly dependent on ethylene and ripening transcription factors, such as RIPENING INHIBITOR (RIN) and COLORLESS NON-RIPENING (CNR), which act as master switches that initiate downstream transcriptional programs (Vrebalov *et al.*, 2002). Key target genes include those encoding photosynthetic proteins (down-regulated), carotenoid biosynthetic enzymes (up-regulated), and ethylene signaling components that trigger the developmental cascade (Fujisawa *et al.*, 2013; Liu et al., 2015; Li *et al.*, 2019; Niu *et al.*, 2025; Wang and de Maagd, 2025). Hormones are central to this network, and ethylene is the primary trigger for ripening in tomato and other climacteric fruits (Liu et al., 2015; T. Sun *et al.*, 2022). These signals reprogram nuclear gene expression toward ripening functions and away from photosynthesis.

### Chlorophylls degrade and carotenoid gene expression changes

Carotenoids are produced from precursors supplied by the methylerythritol 4-phosphate (MEP) pathway, channeled into the carotenoid pathway by phytoene synthase (PSY) and converted into downstream products by other enzymes. Some of these enzymes are encoded by genes specifically expressed in fruit during ripening. In particular, the PSY1 isoform is the main contributor to carotenoid biosynthesis in tomato fruit (Ezquerro *et al.*, 2023). A boost in *PSY1* expression early during ripening contributes to drive the flux of isoprenoid precursors away from chlorophyll and other photosynthetic compounds and into the carotenoid pathway. Furthermore, down-regulated expression of lycopene cyclases prevents conversion of lycopene into downstream carotenoids. The breakdown of chlorophyll governed by the magnesium chelatase product of the *STAY-GREEN* (*SGR*) gene is another key regulatory process. Ripe fruits of the tomato SGR-deficient mutant *green flesh* (*gf*) accumulate carotenoids but fail to fully degrade chlorophyll, resulting in chlorochromoplasts; that is, chromoplasts with thylakoids and grana along with characteristic structures of tomato chromoplasts (Cheung *et al.*, 1993; Barry *et al.*, 2008; Sadali *et al.*, 2019). SGR itself was reported to physically interact with tomato PSY1, presumably resulting in reduced PSY activity (Luo *et al.*, 2013). Interestingly, the DnaJE chaperone ORANGE (OR), considered for a long time as the only molecular switch specifically triggering chromoplast differentiation, interacts with PSY to promote its activity but it also binds and stabilizes the chlorophyll biosynthetic magnesium chelatase subunit CHLI (Zhou *et al.*, 2015; Sun *et al.*, 2023). These interactions suggest a metabolically coordinated regulation of chlorophylls and carotenoids during ripening. Recently, a missense mutation in another magnesium chelatase subunit, CHLH, encoded by the *GENOMES UNCOUPLED 5* (*GUN5*) gene, was found to have an altered ripening phenotype characterized by reduced carotenoid content and delayed chloroplast-to-chromoplast transition (Xu *et al.*, 2025). These results suggest that GUN5-mediated plastid retrograde signaling promotes tomato fruit ripening. An additional mechanism to sense ripening progression appears to result from the ‘self-shade’ effect that chlorophyll causes as light passes through the fruit flesh. Lower amounts of chlorophylls result in a reduced absorption of red light, a feature that causes the degradation of the transcription factor PHYTOCHROME INTERACTING FACTOR 1a (PIF1a), a direct repressor of *PSY1* expression (Llorente *et al.*, 2016). This molecular mechanism is proposed to adjust *PSY1* expression to the actual stage of ripening based on the pigment profile of the fruit at each moment (Llorente *et al.*, 2017).

### Chloroplasts begin to transform into chromoplasts

*In vivo* analysis of chlorophyll and carotenoid content of plastids using confocal microscopy (Egea *et al.*, 2011; D’Andrea *et al.*, 2014; Zheng *et al.*, 2025) demonstrated that chromoplast differentiation starts before chlorophyll is fully lost. At the cellular level, intermediate plastids are observed that still contain chlorophyll and photosynthetic membranes while carotenoids begin to accumulate. This initial transformation of chloroplasts into intermediate plastids sets the stage for the more extensive remodeling of protein import machinery, ultrastructure reorganization, and metabolic reprogramming that follow in subsequent stages.

### Protein import and degradation machineries are reconfigured

Coupled with a significant shift in metabolism, transformation of chloroplasts into chromoplasts involves substantial changes in the fruit proteome (Barsan *et al.*, 2012; Wang *et al.*, 2013). Specifically, there is a coordinated down-regulation of proteins associated with photosynthesis and a significant up-regulation of proteins required for lipid metabolism and carotenoid biosynthesis and sequestration. Most of these changes involve degradation of proteins that were present in chloroplasts and import of new ones into differentiating chromoplasts. The only protein-coding gene in the plastome that is active in chromoplasts, *accD*, is involved in fatty acid biosynthesis (Kahlau and Bock, 2008; Barsan *et al.*, 2012). Turnover of protein translocon components by the chloroplast-associated protein degradation (CHLORAD) ubiquitin–proteasome pathway, including the E3 ligase SP1 and related factors, helps reconfigure the plastid protein import machinery so the organelle imports a chromoplast-appropriate proteome rather than photosynthetic proteins (Ling *et al.*, 2021). Whether ubiquitination also occurs inside plastids to remove unwanted proteins remains controversial (Jarvis *et al.*, 2024). By contrast, the Clp protease complex, the main proteolytic machinery present in the plastid stroma, is required for the chloroplast-to-chromoplast transition (D’Andrea *et al.*, 2018). Moreover, autophagy also controls the degradation of plastid proteins. Autophagosomes containing chloroplast-derived vesicles increase significantly during early fruit ripening (Guo *et al.*, 2025). Interestingly, the OR chaperone plays a role in both protein import and turnover through physical interaction with components of the protein translocon machinery at the inner chloroplast envelope (TIC) to regulate the import of both photosynthetic and non-photosynthetic proteins (Yuan *et al.*, 2021) and with members of the Clp protease complex such as ClpC1 to control PSY proteostasis (Zhou *et al.*, 2015; Welsch *et al.*, 2018).

### Plastid ultrastructure is remodeled

During this stage, thylakoid membranes are progressively dismantled, granal stacking is lost, and internal plastid architecture is reorganized to create new membranous compartments and expand plastoglobules to accommodate increasing levels of newly formed carotenoids. In red ripe tomatoes, lycopene accumulates to high concentrations and precipitates, resulting in crystalloids within membranous sacs, which ultrastructural studies suggest may be derived from the remnants of thylakoid membranes. By contrast, β-carotene is typically found in plastoglobules (Harris and Spurr, 1969; van Wijk and Kessler, 2017). Structural plastoglobule proteins such as fibrillins (FBNs) are essential for assembling the carotenoid sequestration scaffold and stabilizing these plastidial lipid droplets (Deruère *et al.*, 1994; Singh and McNellis, 2011). In tomato, genome-wide studies have identified a family of FBN-encoding genes, with several members showing high expression during fruit ripening (H. Sun *et al.*, 2022; Liu *et al.*, 2024; Almeida *et al.*, 2025). While overexpression of FBN-encoding genes resulted in increased carotenoid content in tomato fruit (Simkin *et al.*, 2007), loss-of-function lines showed aberrant plastoglobule formation and plastid ultrastructure (Almeida *et al.*, 2025), reflecting the involvement of these proteins in creating an effective storage sink. Interestingly, FBN1 protein levels are increased in the carotenoid-overaccumulating fruit of high-pigment tomato mutants *hp1* and *hp2*, which display loss-of-function lesions in the ubiquitin ligase complex components DNA DAMAGE-BINDING PROTEIN1 (DDB1) and DEETIOLATED1 (DET1), respectively (Azari *et al.*, 2010; Kilambi *et al.*, 2013). The fruits of *hp1* and *hp2* mutants were previously found to develop increased size and number of chromoplasts, suggesting that the sequestration and stabilization of carotenoid pigments is coordinated with the control of plastid division during ripening. In agreement, OR was found to interact with FBN1 homologs to promote plastoglobule proliferation and improve carotenoid sequestration (Zhou *et al.*, 2023), and with ACCUMULATION AND REPLICATION OF CHLOROPLASTS 3 (ARC3) to repress the division of chromoplasts, resulting in larger chromoplasts and a higher accumulation of carotenoids (Sun *et al.*, 2020).

### Carotenoid biosynthetic enzymes are recruited to plastoglobules and membrane structures

Proteomic and biochemical work in tomato has shown that plastoglobules and other plastid lipid compartments become enriched in the enzymes that transform MEP-derived precursors into β-carotene (Zita *et al.*, 2022). These enzymes are normally absent from chloroplast plastoglobules, suggesting an effective enzyme-recruiting capacity to the sites where carotenoids accumulate and are sequestered (Ytterberg *et al.*, 2006). This spatial relocalization boosts metabolic flux to carotenoids but also storage efficiency.

### Metabolic reprogramming and heterotrophy

As they complete their conversion, chromoplasts become metabolically heterotrophic, and metabolic networks shift toward producing and storing carotenoids (Egea *et al.*, 2010; Li and Yuan, 2013; Nogueira *et al.*, 2013; Iglesias-Sanchez *et al.*, 2025). Mature chromoplasts lack photosynthetic activity, contain large lycopene crystals and plastoglobules enriched in β-carotene, and have a chromoplast-specific ultrastructure. The transformation is largely irreversible, even though there are reports of tomato ripe fruit regreening under particular conditions (Mesa *et al.*, 2025).

While the described set of events refers to tomato fruit ripening, the main features can be extended to other natural chromoplastogenesis systems (Wang *et al.*, 2013). However, the diversity in carotenoid composition and chromoplast ultrastructure found in nature involves some particularities occurring in particular species. Among them, pepper (*Capsicum annuum*) provides another excellent model for the study of carotenoid accumulation and chloroplast-to-chromoplast differentiation. During ripening, pepper chloroplasts differentiate into chromoplasts that contain fibrillar structures to store large amounts of unique carotenoids such as capsanthin and capsorubin (Rödiger *et al.*, 2021; Berry *et al.*, 2022). Coordinated shifts in transcriptomic and proteomic profiles are again central to pepper fruit ripening, involving the up-regulation of carotenoid biosynthetic genes alongside the down-regulation of photosynthetic genes (Kim *et al.*, 2018; Rödiger *et al.*, 2021). In particular, FBNs are also involved in pepper chromoplastogenesis, but instead of plastoglobules they form the structural backbone of the characteristic fibrils found in the chromoplasts of this species (Kilcrease *et al.*, 2015). These results illustrate that while the overarching theme of converting chloroplasts to chromoplasts is conserved, different plant lineages have evolved slightly different ultrastructural and regulatory solutions.

## Synthetic plastids as models

Despite the plethora of studies addressing the differentiation of chloroplasts into chromoplasts and other plastid types, until recently we could not answer a relatively simple question: do chloroplasts de-differentiate in response to a programmed developmental signal or as an adaptive response to overaccumulation of particular metabolites (e.g. carotenoids in the case of chromoplasts). In other words, what came first, the differentiation of chromoplasts or the accumulation of carotenoids? This question has been conclusively answered recently thanks to the use of genetic and chemical tools that artificially transform leaf chloroplasts into chromoplasts and tocopherol-enriched plastids, providing experimental systems in which chloroplast conversion into storage organelles is not linked to a developmental program such as fruit ripening (e.g. in tomato) or secondary root growth (e.g. in carrot). In this section we will review what we have learned from these artificial systems about the molecular basis of plastid differentiation.

Overexpression of a PSY-encoding gene in dark-grown calli and roots of the model plant *Arabidopsis thaliana*, a species that does not naturally develop chromoplasts, was found to be sufficient for the differentiation of chromoplast-like plastids harboring carotenoid crystals (Maass *et al.*, 2009; Schaub *et al.*, 2018). This process only occurred in lines that overproduced phytoene beyond a certain threshold (Schaub *et al.*, 2018), suggesting that a strong enough activation of PSY activity could by itself trigger the differentiation of chromoplasts, at least in non-green tissues. More recently, synthetic chromoplasts were produced in leaves of several plant species (including *A. thaliana*) by overexpressing a bacterial PSY enzyme referred to as crtB (Llorente *et al.*, 2020; Morelli *et al.*, 2023, 2024). The observation that boosting phytoene biosynthesis was sufficient to trigger the overaccumulation of carotenoids and promote chromoplast differentiation in tissues or species that do not normally develop these plastid types represents strong evidence that chromoplastogenesis does not require a specific developmental program but it is most likely to be a response triggered by the need to store highly lipophilic compounds in a safe location. Supporting this conclusion, it has been shown that the production of high carotenoid levels in the cytosol of plant cells (a location where carotenoids are not naturally found) resulted in the formation of storage structures very similar to those typical of chromoplasts, including large lipid droplets resembling plastoglobules and undulating membranes like those found in chromoplasts harboring carotenoid crystals (Morelli *et al.*, 2024).

**Fig. 3.**
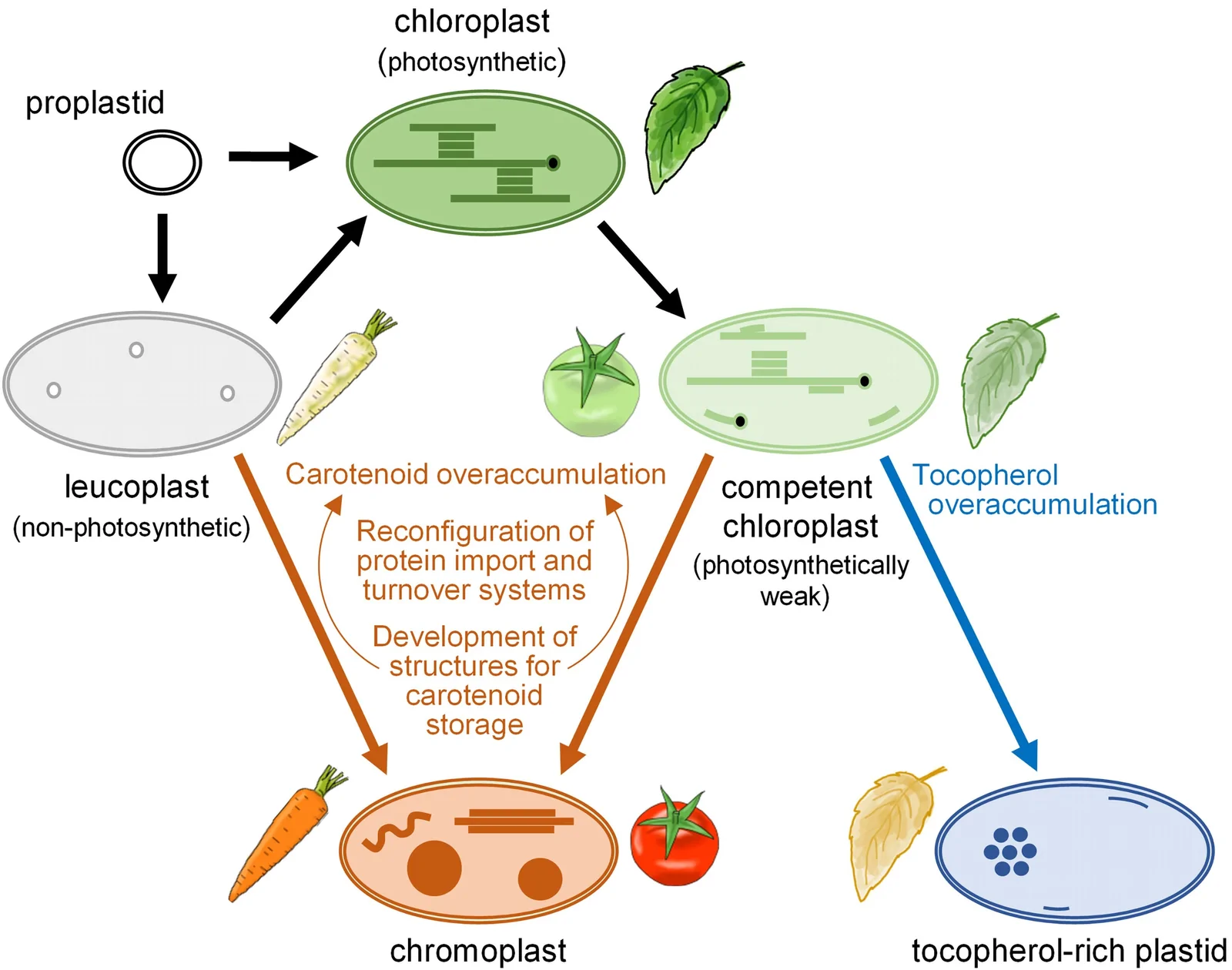
Model of plastid transition stages. Proplastids develop into chloroplasts in leaves or into other plastid types such as leucoplasts in carrot roots, which may eventually convert into chloroplasts under certain environmental conditions (e.g. in the light). Genetic, environmental, or metabolic cues can lead to chloroplasts with a marginal photosynthetic activity (e.g. those in mature green tomatoes). Loss or reduction of chloroplast identity is the first requisite for their conversion into other plastid types. The second requirement is a sufficient buildup of particular metabolites, and the nature of these metabolites determines the final ultrastructure of the plastids. In the case of chromoplasts, the accumulation of carotenoids reconfigures protein import and turnover systems for the eventual development of sequestering structures such as plastoglobules or membrane sacs, which in turn favor carotenoid synthesis and storage. Tocopherol-rich plastids characterized by clusters of small plastoglobules develop in leaves treated with the synthetic compound X57, which weakens chloroplast identity and promotes tocopherol accumulation by different pathways.

Transplastomic tobacco plants expressing a full pathway for the production of astaxanthin, a non-plant carotenoid, also showed a conversion of leaf chloroplasts into synthetic chromoplasts with proliferated plastoglobules and remodeled membrane systems (Lu *et al.*, 2017). This result demonstrates that it is not the specific accumulation of phytoene that triggers the chromoplastogenesis process. Instead, overproducing carotenoids normally absent from chloroplasts (such as phytoene in *crtB*-transformed leaves or astaxanthin in transplastomic plants) probably interferes with the photosynthetic machinery, causing chloroplasts to lose functionality and hence become competent for conversion into chromoplasts (Llorente *et al.*, 2020). The view that a loss or reduction in photosynthetic activity is a requirement for chloroplast-to-chromoplast transformation rather than just a consequence of launching a presumed differentiation program has been a major paradigm change. Based on these results, a two-step model was proposed for chloroplast-to-chromoplast differentiation, in which the first step is a loss of chloroplast identity (i.e. a reduction or removal of photosynthetic activity). This initial step is not necessary when chromoplasts differentiate from non-photosynthetic plastids (such as those of young carrot roots) or even for chloroplasts with naturally low photosynthetic competence (such as those of mature green tomato fruits) (Fig. 3). Once plastids become competent (i.e. display low photosynthetic capacity), a sufficiently increased production of carotenoids is all that is needed for the differentiation of chromoplasts in a second step that involves a massive reprogramming of nuclear gene expression, plastid proteome and metabolome changes, and ultrastructural rearrangements similar to those taking place during natural chromoplastogenesis (Llorente *et al.*, 2020; Morelli *et al.*, 2023) (Fig. 3).

Recent results suggest that this basic scheme for the transformation of chloroplasts into storage compartments might also apply to all or most plastid conversions (Fig. 3). Induction of tocopherol overaccumulation in leaves treated with a chemical compound named X57 transformed chloroplasts into a new plastid type with dismantled photosynthetic membranes, reduced levels of chlorophylls and carotenoids, and a proliferation of clusters of small plastoglobules (Perez-Colao *et al.*, 2026). Interestingly, this process is reversible (Perez-Colao, Cruces *et al.*, 2026), which makes X57 a unique chemical tool that should allow key molecular players driving chloroplast transitions to be unveiled. The use of X57 has allowed the definition of a new molecular cascade involving the SAL1–PAP retrograde pathway for the weakening of chloroplast identity in the first step of the conversion process. In a second stage, X57 treatment leads to the development of plastoglobule-rich plastids by triggering tocopherol overproduction by a SAL1/PAP-independent mechanism (Fig. 3). Besides supporting the two-step model for chloroplast conversions, these results offer novel experimental evidence that plastid differentiation is not the result of a terminally fixed developmental commitment but a dynamic adaptive response to the need for sequestering particular molecules.

While the first step of chromoplastogenesis is not a requisite when starting from plastids showing no or low photosynthetic activity, the second step (i.e. an induction of carotenoid biosynthesis) was recently found to also be dispensable in some cases (Zheng *et al.*, 2025). Transient overexpression of the pepper (*C. annuum*) *SGR1* gene (encoding a magnesium dechelatase) in leaves triggered chlorophyll degradation and disassembly of photosynthetic pigment–protein complexes. In the dark [i.e. when the production of toxic reactive oxygen species (ROS) was prevented], carotenoids were released from dismantled complexes and sequestered into plastoglobules and crystalloid structures, forming chromoplast-like plastids that were referred to as chromogerontoplasts (Fig. 1E). Together, the available lines of evidence allow a new definition of chromoplasts to be proposed that includes those that naturally develop in some plant tissues as well as those engineered in leaves by manipulating carotenoid biosynthesis and/or storage pathways (Box 1).

## Mechanistic insights

Our knowledge of the molecular mechanisms directly regulating plastid conversions is quite limited, in part because transitions involve a multitude of pathways. As mentioned above, several mechanisms can result in chloroplasts becoming competent for transformation into storage plastids, whereas the second phase of the transformation will depend on diverse parameters, including the chemical nature of the overproduced metabolite, the tissue, or the plant species.

In nature (e.g. in tomato fruit), developmental cues (i.e. ripening) are likely to be responsible for repressing photosynthetic function, making mature green fruit chloroplasts competent in the first step of the chloroplast-to-chromoplast transformation process. However, environmental inputs can also be important. For example, oxidative stress resulting from water shortage or excess light might trigger a SAL1-dependent response to further weaken chloroplast function. Ripening-related developmental regulators are also responsible for altering the expression of carotenoid biosynthetic genes (e.g. up-regulating *PSY1* expression) at the breaker stage, hence fueling the second step of the chromoplast differentiation process. In this second phase, retrograde signals produced by challenged chloroplasts and differentiating plastids probably support the organellar transformation by reprogramming nuclear gene expression and whole-cell metabolism. Some of these signals are presumably similar in all plastid differentiation processes (e.g. those related to the mechanical or chemical constraints derived from accommodating large amounts of carotenoids, tocopherols, or other lipophilic metabolites), while others might vary between chromoplasts of different species or tissues that accumulate different types of carotenoids (e.g. lycopene is stored as crystals but xanthophylls typically accumulate in plastoglobules).

Apart from the proposed contribution of the *GUN5*-dependent pathway in tomato fruit (Xu *et al.*, 2025), the identity of the retrograde signals involved in chromoplastogenesis remains unknown. *Arabidopsis thaliana* mutants defective in different retrograde pathways such as *gun1*, *sal1*, and *csb3* showed no significant changes compared with wild-type controls in terms of crtB-mediated chromoplastogenesis (Morelli *et al.*, 2024), By contrast, the X57-triggered formation of plastoglobule-rich plastids was abolished in *sal1* plants because this compound was unable to weaken chloroplast identity in the absence of a functional SAL1/PAP pathway. *Arabidopsis thaliana* mutants were also used to investigate the possible contribution of apocarotenoids, signaling molecules derived from the oxygenic cleavage of carotenoids (Li *et al.*, 2025). Plants lacking the plastidial carotenoid cleavage dioxygenases CCD1 and CCD4 showed no defects in the crtB-triggered transformation of chloroplasts into chromoplasts (Llorente *et al.*, 2020). However, it cannot be excluded that the differentiation process could be regulated by apocarotenoids that might be formed by the activity of other enzymes or even non-enzymatically, such as by ROS. Indeed, ROS resulting from the disruption of the photosynthetic apparatus, as well as the associated redox changes, were found to promote the crtB-dependent chromoplast differentiation process (Morelli *et al.*, 2023). Apocarotenoids include retrograde signals such as β-cyclocitral and hormones such as strigolactones and abscisic acid (ABA). While the possible involvement of β-cyclocitral has not been tested yet, exogenous treatment with hormones has shown that the synthetic leaf chromoplastogenesis process is accelerated by the synthetic strigolactone GR24, whereas ABA shows no effect (Morelli *et al.*, 2024). By contrast, both GR24 and ABA promote carotenoid biosynthesis and chromoplast differentiation in tomato fruit (Galpaz *et al.*, 2008; Zhang *et al.*, 2009; Naeem *et al.*, 2025). How chromoplast differentiation might be modulated by other apocarotenoids involved in chloroplast developmental transitions, including those derived from linear carotenoids such as phytoene (León *et al.*, 2025), remains unknown.

## Conclusions

Plastids dynamically interconvert, creating intermediate organelles and specialized types that store proteins and metabolites. In particular, carotenoid-accumulating chromoplasts are relevant for the nutritional and organoleptic quality of many fruits. Recent advances, especially those derived from the synthetic differentiation of chromoplast-like plastids from leaf chloroplasts, have greatly contributed to more precisely defining what a chromoplast is (Box 1) and to better understand how a chromoplast is made (Fig. 2). From these studies, it has become clear that chromoplastogenesis requires extensive rewiring of nuclear gene expression to support major changes in the organellar proteome, metabolome, and architecture. While we still have many knowledge gaps and open questions (Box 2), the available data suggest that the metabolic cues are the main drivers of the conversion of chloroplasts into storage plastids. Indeed, the results showing that enriching competent chloroplasts in particular metabolites can trigger their conversion *per se* demonstrate the outstanding ability of plastids to adapt their ultrastructure to accommodate diverse compounds under very different developmental and environmental contexts. Furthermore, they raise the question of how specific metabolic perturbations differentially program retrograde signaling, eventually controlling nuclear genes required for protein import and turnover, membrane remodeling, and metabolic readjustments (Nogueira *et al.*, 2013; Llorente *et al.*, 2020; Perez-Colao, Cruces *et al.*, 2026; Zheng *et al.*, 2025). Unveiling the molecular pathways sensing the unbalanced accumulation of particular metabolites and transducing the signal into the regulation of specific sets of nuclear genes (e.g. those required to construct the structures that will sequester the overproduced metabolite) remains an important challenge for the future.

Box 2. **Knowledge gaps and open questions**

- What are the regulatory mechanisms that maintain chloroplast identity when challenged and prevent their transformation into storage plastids?
- Are there common molecular players orchestrating the first steps of the conversion of competent chloroplasts into different plastid types?
- How do metabolic perturbations differentially shape nuclear programs to eventually support ultrastructural changes associated with particular plastid types?
- What are the retrograde pathways involved in the differentiation process? Are apocarotenoids involved in chromoplastogenesis or/and other plastid transitions?
- What is the molecular footprint of intermediate plastid types (chlorochromoplasts, etc.)?
- Can a quorum-sensing mechanism explain the presence in the same cell of plastids in several differentiation stages? What might be the chemical signal(s) involved?
- How can we apply the current knowledge for enhanced biofortification? Can our current molecular tools be optimized for a custom-made design of storage plastids in particular tissues or organs of interest?
